# Molecular Insights of Genetic Variation in *Erianthus arundinaceus* Populations Native to China

**DOI:** 10.1371/journal.pone.0080388

**Published:** 2013-11-25

**Authors:** Jianbo Zhang, Jiajun Yan, Yunwei Zhang, Xiao Ma, Shiqie Bai, Yanqi Wu, Zhixue Dao, Daxu Li, Changbing Zhang, Yu Zhang, Minghong You, Fuyu Yang, Jin Zhang

**Affiliations:** 1 Department of Grassland Science, Animal Science and Technology College, Sichuan Agricultural University, Ya'an, Sichuan, China; 2 Sichuan Academy of Grassland Science, Chengdu, Sichuan, China; 3 Guizhou Grassland Science Institute, Guiyang, Guizhou, China; 4 Grassland Institute, China Agricultural University, Beijing, China; 5 Plant and Soil Sciences Department, Oklahoma State University, Stillwater, Oklahoma, United States of America; Tulane University Health Sciences Center, United States of America

## Abstract

**Background:**

*E. arundinaceus* (Retz.) Jeswiet is a warm-season, tall-growing perennial species native to much southern portion in China. The grass has been extensively used in sugarcane breeding and is recently targeted as a bioenergy feedstock crop. However, information on the genetic structure of the Chinese wild germplasm is limited. Knowledge of genetic variation within and among populations is essential for breeding new cultivars in the species. The major objective of this study was to quantify the magnitude of genetic variation among and within natural populations in China.

**Methodology/Principal Findings:**

In this experiment, we analyzed genetic variation of 164 individuals of 18 populations collected from natural habitats in six Chinese provinces using 20 sequence-related amplified polymorphism (SRAP) primer pairs generating 277 polymorphic bands. Among and within the populations, the percentage of polymorphic bands (*PPB*) was 80.00% and 27.07%, genetic diversity (*H_E_*) was 0.245 and 0.099, effective number of alleles (*N_E_*) was 1.350 and 1.170, and Shannon's information index (*I*) was 0.340 and 0.147, respectively. The populations were clustered into six groups exhibiting a high level of genetic differentiation, which was highly associated with geographic origins of respective germplasm populations, but was not significantly associated with geographic distances between the populations.

**Conclusions/Significance:**

This is the first report indicating that large genetic variation exists in the Chinese *E. arundinaceus* germplasm based on the SRAP molecular marker analysis of native populations. The genetic structure of populations in the species has been substantially affected by geographic landforms and environments. The diverse collection will be highly valuable in genetic improvement in the species per se and likely in sugarcane.

## Introduction

*E. arundinaceus* (synonym of *Saccharum arundinaceum Retz*.) is a warm-season, tall-growing, caespitose perennial species native to China and certain other south and southeast Asian nations of temperate climates to tropical environments [1]–[2]. As a wild relative of sugarcane (*Saccharum officinarum* L.), the species has contributed to the genetic improvement in sugarcane breeding [3] and possesses high potential for the development of energy cane interspecific hybrids [4]. It is widely distributed in the Chinese provinces of Anhui, Fujian, Guangdong, Guangxi, Guizhou, Hainan, Henan, Hubei, Hunan, Jiangsu, Jiangxi, Shanxi, Sichuan, Taiwan, Xinjiang, Xizang, Yunnan, and Zhejiang [5]. The species is related to taxa in *Miscanthus*, *Narenga*, *Saccharum*, and *Sclerostachya*, so is considered to be a member of the “sugarcane complex” [6]. Due to its excellent tolerance to abiotic stresses and disease resistance, the species has long been used in sugarcane breeding [7]. Although difficult, breeders have successfully generated fertile *Saccharum* × *Erianthus* hybrids, which are further crossed to sugarcane clones in the production of wide intergeneric hybrids [8]–[11]. Recently, the species has been targeted as a bioenergy perennial because of its high biomass yield potential on marginal lands [12]. With the support from the National High-Tech R&D Program of China, a breeding program has been initiated to improve the species as a bioenergy feedstock crop at the Sichuan Academy of Grassland Science, China since 2011.

Genetic variation in *E. arundinaceus* has been well documented. Using morphological traits, a high level of variation was reported in *E. arundinaceus* accessions from China, while the variation from Indonesia was relatively low [13]–[14]. Karyotype analyses indicated most clones of Chinese *E. arundinaceus* had 2n = 4x = 40 and 6x = 60 somatic chromosomes while 2n  = 2x = 20 was rare [15]. Using DNA markers, the percentage of polymorphic bands ranged from 65 to 99% indicating high molecular diversity in Chinese germplasm [16]–[18], while *E. arundinaceus* from Indonesia appeared to have a low level of molecular variability [18]–[20]. *E. arundinaceus* from India was more polymorphic than from Indonesia [18], [21]. Although useful, these reports revealed very limited information on genetic variation among and within populations in the species.

In the last two decades, amplified fragment length polymorphism (AFLP), inter simple sequence repeat (ISSR), random amplified polymorphic DNA (RAPD), restriction fragment length polymorphism (RFLP) but sequence-related amplified polymorphism (SRAP) have been used in characterizing genetic diversity in *E. arundinaceus* [16]–[20]. SRAP has been proved to be a reliable molecular marker system based on simple PCR amplifications of genomic DNA [22]. The marker system analyzes DNA polymorphisms with amplifying open reading frames using specifically designed primers. SRAP markers provide a valuable tool to study patterns of genetic variability due to their advantages over other molecular markers, such as less complex and labor-saving procedures and more random sampling of the whole genome.

Information on genetic variation among and within populations could help better understand the natural variation in the species on a large geographic scale, which is useful in sampling and deploying the germplasm in breeding programs. We collected 18 indigenous populations of *E. arundinaceus* in six provinces of China. Therefore, the major objective of this study was to quantify the magnitude of genetic variation among and within the natural populations.

## Materials and Methods

### Ethics Statement

This study was approved by the Department of Grassland Science, Animal Science and Technology College, Sichuan Agricultural University; Sichuan Academy of Grassland Science; Guizhou Grassland Science Institute; and Grassland Institute, China Agricultural University. No specific permissions were required for collecting *Erianthus arundinaceus* samples at the locations in China, because the research was funded by the Ministry of Science and Technology and the earmarked fund for China Agriculture Research System of the People's Republic of China, and the species is not an endangered or protected species.

### Sample Collection and DNA Extraction

**Figure 1:**
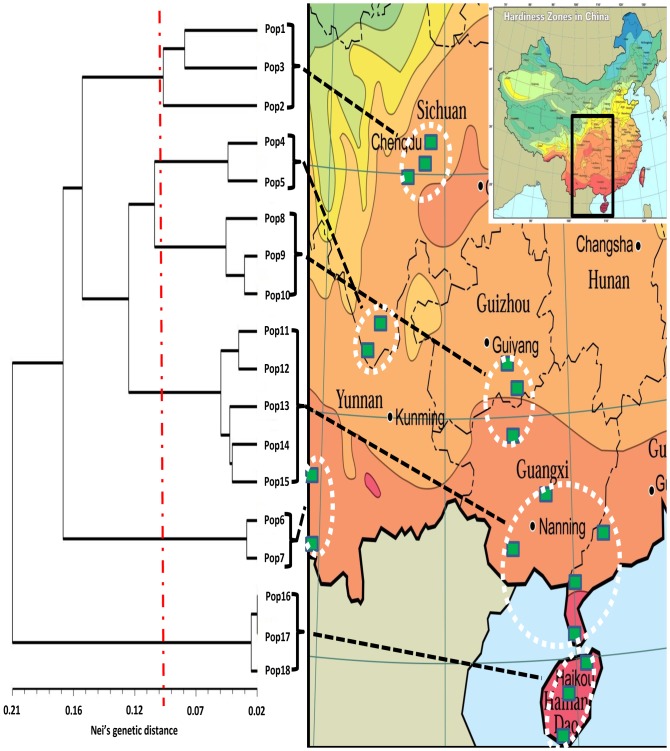
UPGMA phenogram illustrating genetic relationships among 18 populations of *E. arundinaceus*, based on Nei's (1978) genetic distances calculated from 294 polymorphic bands. Numbers on branches indicate bootstrap values with 1000 replicates.

Following the population sampling method by Jing and Lu [23], a total of 164 wild *E. arundinaceus* individual leaf samples in 18 populations were collected in Sichuan, Yunnan, Guizhou, Guangxi, Guangdong and Hainan provinces, China (see Table 1). Sampled individuals in each population ranged from six to 10. Localities of the collected populations spanned nearly 14°N. latitudes from tropical environments in Hainan to subtropical climates in Sichuan (Table 1 and Figure 1). The leaf tissues were dried using self-indicating silica gel and stored in a freezer at −80°C until DNA extraction. Genomic DNA was isolated using the modified CTAB method of Doyle [24]. Purity and concentration of the genomic DNA were determined with a Nanodrop spectrophotometer (NanoDrop Products, Wilmington, DE).

**Table 1:** Population designation, location, altitude, latitude, longitude, habitat and sample size per population in each sampling site.

Population designation	County/city, Province	Altitude (m)	Latitude (N.)	Longitude (E.)	Habitat	Sample size
Pop1	Longquanyi, Sichuan	801	32°32′	104°20′	shrub slope	10
Pop2	Leshan, Sichuan	355	29°33′	103°47′	shrub slope	10
Pop3	Meishan, Sichuan	419	30°02′	103°46′	roadside	10
Pop4	Huili, Sichuan	1743	26°38′	102°15′	shrub slope	10
Pop5	Ningnan, Sichuan	694	26°59′	102°48′	riverside	10
Pop6	Menglian, Yunnan	1111	22°52′	099°48′	roadside	7
Pop7	Shuangjiang, Yunnan	887	23°02′	099°49′	roadside	6
Pop8	Dushan, Guizhou	944	25°46′	107°34′	field edge	10
Pop9	Rongjiang, Guizhou	235	25°56′	108°32′	riverside	10
Pop10	Hechi, Guangxi	284	24°39′	107°52′	roadside	10
Pop11	Nanning, Guangxi	90	22°38′	108°23′	roadside	10
Pop12	Sanjiang, Guangxi	168	25°47′	109°39′	riverside	10
Pop13	Suixi, Guangdong	43	21°33′	110°01′	roadside	7
Pop14	Xuwen, Guangdong	9	20°55′	110°04′	field edge	10
Pop15	Xinyi, Guangdong	109	22°20′	110°55′	roadside	10
Pop16	Sanya, Hainan	30	18°34′	109°37′	wasteland	9
Pop17	Danzhou, Hainan	20	19°43′	109°27′	wasteland	6
Pop18	Wuzhishan, Hainan	214	18°59′	109°34′	shrub slope	9

### SRAP Amplification

Sequence related amplified polymorphism analysis was conducted according to a previously established protocol [22]. Twenty primer pairs (PPs) were selected from 120 available PPs. The PPs were synthesized by Shanghai Biochemical Engineering Technology (Shanghai, China). PCR reactions were performed in 20 µL reactions containing 1 µL 2 µg/µL DNA, 12.5 µL 2× Reaction Mix (Tiangen Beijing, China), 0.2 µL (units) Golden DNA Polymerase (Tiangen Beijing, China), 1 µL 10 mM forward primer, 1 µL 10 mM reverse primer, and 4.3 µL of sterile water. PCR amplification reactions were performed in a Mastercycler Pro (Eppendorf, Germany) under the following thermal conditions: 5 min at 94°C; 5 cycles of 94°C,1 min; 35°C, 1 min; and 72°C, 2 min; additional 35 cycles of 94°C, 1 min; 50°C, 1 min; and 72°C, 1 min; extension of 5 min at 72°C; and a final storage at 4°C. Products in PCR reactions were separated using 6% denatured polyacrylamide gels [acrylamide-bisacrylamide (19∶1), 1.0×TBE]. After electrophoreses, gels were stained in a AgNO_3_ solution. Gel images were then photographed by Gel Doc(TM) XR System (Bio-Rad, USA).

### Data Analysis

Clearly amplified PCR bands were visually scored for presence (1) or absence (0), and then were assembled into an Excel matrix for the following analyses. Use of dominant marker data in genetic diversity analysis can lead to estimation bias with overestimating parameters by as much as 5%, especially with small sample sizes [25]–[26]. To account for this potential bias, Lynch and Milligan proposed pruning any locus with a band frequency of higher than 1-(3/N), where N is the number of individual samples [25]. Since SRAP markers are dominant, only the marker data of specific loci having a band frequency less than 1-(3/164) = 0.982 were retained for subsequent statistical analyses in this study.

The number of polymorphic loci (*Np*), percentage of polymorphic bands (*PPB*), Shannon's information index (*I*), observed number of alleles(*N_O_*), effective number of alleles (*N_E_*), Nei's gene diversity(*H_E_*), genetic diversity within populations (*Hs*), total genetic diversity (*Ht*), genetic differentiation coefficient (*Gst*), gene flow estimates (*Nm*), and Nei's genetic distance were calculated using POPGENE [27]. A UPGMA tree based on Nei's [28] genetic distance data was generated by TFPGA (version 1.3) [29] to examine genetic relationships of the populations while a UPGMA tree among individuals was generated by FreeTree program [30]. Bootstrap values were obtained by resampling replacements over loci in 1000 replicates. In addition, a Mantel test was conducted to calculate the correlation between pairwise geographic and Nei's genetic distances using NTSYS software [31]. Finally, WINAMOVA program v.1.55 [32] was used to separate the total genetic variance into within and among populations/groups. The input files for POPGENE and AMOVA were prepared with the aid of DCFA1.1 program [33].

## Results

Twenty selected SRAP PPs yielded a total of 365 scorable bands, of which 294 were polymorphic (Appendix S1). Using the method by Lynch and Milligan [25], five loci that each was scored more than 161 of “0”, were excluded, while 12 loci with each scored more than 161 of “1”, were changed to monomorphic loci, resulting in 360 scorable and 277 polymorphic bands used in subsequent analyses. The number of amplified bands for each PP ranged from 14 to 22, with an average of 18 bands (Table 2). The percentage of polymorphic bands (*PPB*) within each population ranged from 16.94% (Pop3) to 33.33% (Pop4) with an average of 27.07% while PPB was 80.00% at the species level. Among these 18 populations, Pop4 and Pop14 exhibited the greatest level of variability (*N_O_* = 1.33 and 1.33, *N_E_* = 1.21 and 1.22, *I* = 0.179 and 0.181, and *H_E_* = 0.121 and 0.123, respectively). By contrast, genetic diversity was the least in Pop3, with *N_O_* = 1.17, *N_E_* = 1.11, *I* = 0.092, and *H_E_* = 0.063. The average of *N_O_*, *N_E_*, *I* and *H_E_* was 1.27, 1.17, 0.147 and 0.099 within populations, and was 1.80, 1.35, 0.340 and 0.245 among the populations, respectively (Table 3).

**Table 3:** Genetic diversity indices for 18 *E. arundinaceus* populations collected in China.

Population	*Np*	*PPB* (%)	*N_O_*	*N_E_*	*I*	*H_E_*
Pop1	110	30.56	1.31	1.20	0.171	0.116
Pop2	67	18.61	1.19	1.12	0.101	0.069
Pop3	61	16.94	1.17	1.11	0.092	0.063
Pop4	120	33.33	1.33	1.21	0.179	0.121
Pop5	89	24.72	1.25	1.16	0.135	0.091
Pop6	87	24.17	1.24	1.17	0.141	0.097
Pop7	89	24.72	1.25	1.18	0.142	0.098
Pop8	113	31.39	1.31	1.19	0.163	0.109
Pop9	108	30.00	1.30	1.18	0.159	0.107
Pop10	102	28.33	1.28	1.17	0.149	0.100
Pop11	113	31.39	1.31	1.18	0.159	0.105
Pop12	119	33.06	1.33	1.21	0.177	0.119
Pop13	101	28.06	1.28	1.19	0.156	0.106
Pop14	119	33.06	1.33	1.22	0.181	0.123
Pop15	108	30.00	1.30	1.18	0.160	0.107
Pop16	81	22.50	1.23	1.14	0.121	0.082
Pop17	74	20.56	1.21	1.15	0.119	0.081
Pop18	93	25.83	1.26	1.16	0.136	0.091
Mean	97	27.07	1.27	1.17	0.147	0.099
Species	288	80.00	1.80	1.35	0.340	0.245

*N*p  =  polymorphic loci; *PPB*  =  percentage of polymorphic loci; *N*_O_  =  number of alleles per locus; *N*_E_  =  effective number of alleles per locus; *I*  =  Shannon's information index; *H*_E_  =  Nei's (1973) measure of gene diversity.

**Table:** Table 2. Twenty SRAP primer pair ID, sequences, amplified bands and percent polymorphic bands.

Primer pair ID	Forward (f) and reverse (r) primer sequences (5′→3′)	Total bands	Polymorphic bands	Percent polymorphic Bands(%)
1f4r	f:TGAGTCCAAACCGGATA r:GACTGCGTACGAATTTGA	22	20	91
1f8r	f:TGAGTCCAAACCGGATA r:GACTGCGTACGAATTCTG	16	11	69
2f8r	f:TGAGTCCAAACCGGAGC r:GACTGCGTACGAATTCTG	14	9	64
2f10r	f:TGAGTCCAAACCGGAGC r:GACTGCGTACGAATTCAG	21	16	76
3f6r	f:TGAGTCCAAACCGGAAT r:GACTGCGTACGAATTGCA	18	12	67
3f9r	f:TGAGTCCAAACCGGAAT r:TGAGTCCAAACCGGTAG	15	13	87
5f5r	f:TGAGTCCAAACCGGAAG r:GACTGCGTACGAATTAAC	15	12	80
6f1r	f:TGAGTCCAAACCGGTAA r:GACTGCGTACGAATTAAT	21	16	76
6f7r	f:TGAGTCCAAACCGGTAA r:GACTGCGTACGAATTCAA	16	9	56
7f8r	f:TGAGTCCAAACCGGTCC r:GACTGCGTACGAATTCTG	15	12	80
7f10r	f:TGAGTCCAAACCGGTCC r:GACTGCGTACGAATTCAG	19	13	68
8f4r	f:TGAGTCCAAACCGGTGC r:GACTGCGTACGAATTTGA	14	11	79
8f7r	f:TGAGTCCAAACCGGTGC r:GACTGCGTACGAATTCAA	19	17	89
9f1r	f:TGAGTCCAAACCGGTAG r:GACTGCGTACGAATTAAT	22	16	73
9f3r	f:TGAGTCCAAACCGGTAG r:GACTGCGTACGAATTGAC	19	15	79
9f8r	f:TGAGTCCAAACCGGTAG r:GACTGCGTACGAATTCTG	21	18	86
10f7r	f:TGAGTCCAAACCGGTTG r:GACTGCGTACGAATTCAA	16	12	75
10f10r	f:TGAGTCCAAACCGGTTG r:GACTGCGTACGAATTCAG	18	13	72
11f1r	f:TGAGTCCAAACCGGTGT r:GACTGCGTACGAATTAAT	22	18	82
11f8r	f:TGAGTCCAAACCGGTGT r:GACTGCGTACGAATTCTG	17	14	82
Mean		18	14	77
Total		360	277	

### Genetic Distance and Phylogenetic Relationship

**Figure 2:**
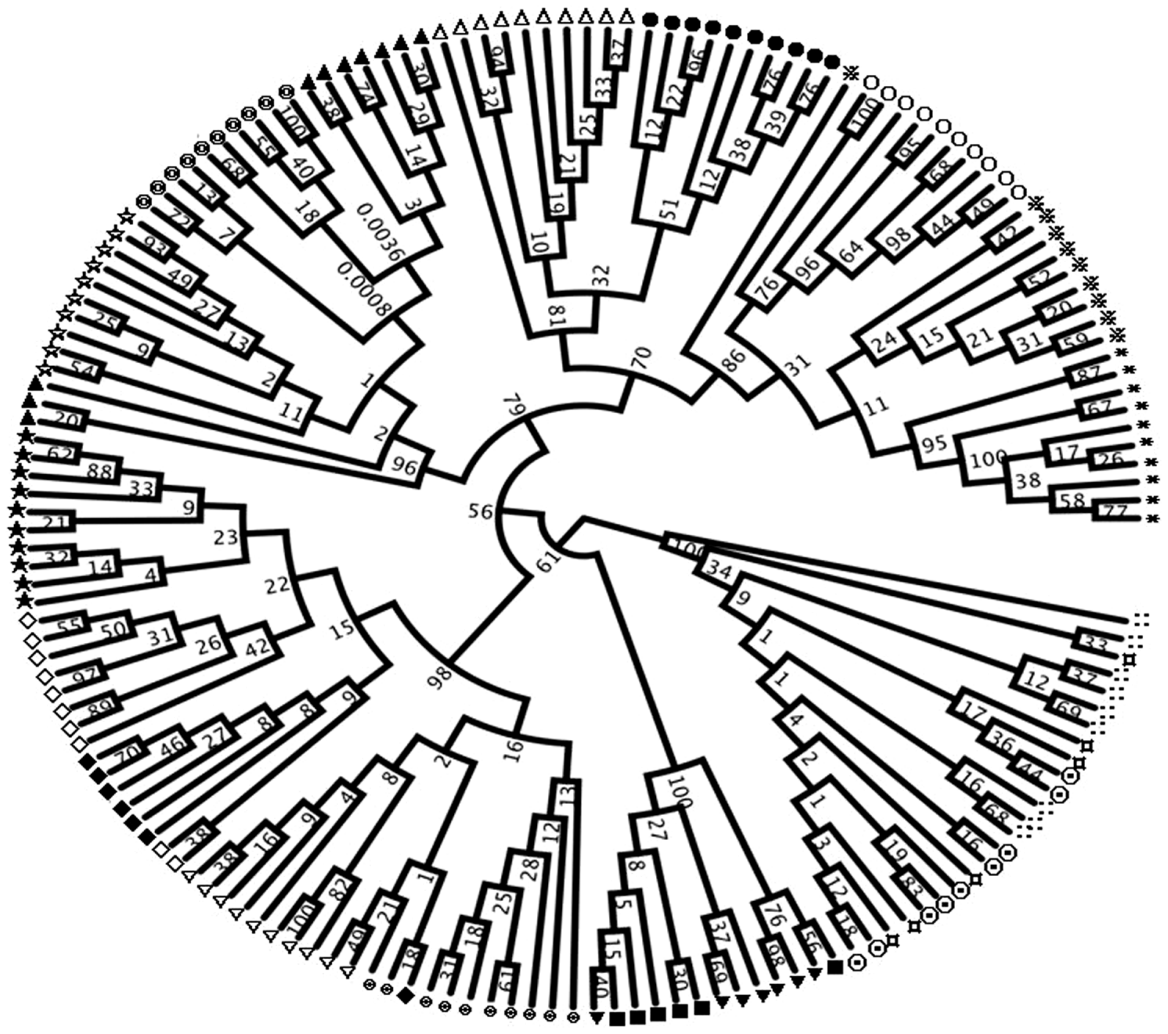
UPGMA cluster analysis based on Nei's (1978) genetic distances among individuals. Numbers on branches indicate bootstrap values from 1000 replicates. Symbols represent populations in the cluster tree as 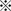 Pop1, ○ Pop2, * Pop3, △ Pop4, • Pop5, ▾ Pop6, ▪ Pop7, 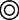 Pop8, ▴ Pop9, ☆ Pop10, ⊕ Pop11, ▽ Pop12, ⧫ Pop13, ◊Pop14, ★ Pop15, ⊙ Pop16, ¤ Pop17, 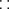 Pop18.

Genetic distances (D, Nei's measure) among populations are given in Table 4. D values ranged from 0.022 (between Pop16 and Pop17) to 0.332 (between Pop3 and Pop18) with an average of 0.154 in the collected germplasm. The UPGMA tree (Figure1) based on the D values among populations revealed that the 18 populations were clustered into six groups. Group 1 included Pop1, Pop2 and Pop3 from the Sichuan Basin. Group 2 encompassed Pop4 and Pop5 from Daliangshan region of Sichuan province. Group 3 consisted of Pop8, Pop9 and Pop10 from Guizhou province except Pop10. Group 4 was the largest group including Pop11, Pop12, Pop13, Pop14 and Pop15 from Guangxi and Guangdong provinces. Group 5 contained Pop6 and Pop7 both from Yunnan province. Group 6 possessed Pop16, Pop17 and Pop18 from Hainan. The UPGMA tree among individuals revealed that 164 individuals were grouped into six clusters (Figure 2) supported by bootstrap values ranging from 0.81 to 1.00. The result was basically consistent with that of UPGMA analysis among populations. Figure 2 indicates individuals from the same populations were almost clustered into the same subgroups with a few exceptions. One individual of Pop1 and three individuals of Pop9 were separated into subgroups different from other individuals in the same populations. Similarly, two individuals of Pop14 were clustered into the same group with individuals of Pop13, and two individuals of Pop12 and two individuals of Pop11 and one individual of Pop13 were clustered into the same subgroup. Individuals of Pop16, Pop17 and Pop18 from Hainan province were clustered into two subgroups. The Mantel tests indicated that there was no significant relationship between genetic distance and geographic distance among populations (r = 0.77, p = 1. 000).

**Table 4:** Estimates of Nei's (1978) unbiased genetic distance between *E. arundinaceus* populations.

	Pop1	Pop2	Pop3	Pop4	Pop5	Pop6	Pop7	Pop8	Pop9	Pop10	Pop11	Pop12	Pop13	Pop14	Pop15	Pop16	Pop17
**Pop2**	0.082																
**Pop3**	0.078	0.109															
**Pop4**	0.079	0.124	0.131														
**Pop5**	0.078	0.129	0.118	0.046													
**Pop6**	0.162	0.225	0.229	0.157	0.140												
**Pop7**	0.167	0.232	0.235	0.162	0.144	0.030											
**Pop8**	0.104	0.151	0.142	0.109	0.100	0.152	0.152										
**Pop9**	0.100	0.154	0.143	0.103	0.101	0.159	0.150	0.039									
**Pop10**	0.100	0.164	0.155	0.100	0.104	0.154	0.148	0.052	0.032								
**Pop11**	0.159	0.215	0.205	0.129	0.128	0.178	0.181	0.131	0.112	0.114							
**Pop12**	0.162	0.210	0.220	0.134	0.141	0.180	0.180	0.126	0.096	0.101	0.039						
**Pop13**	0.143	0.195	0.208	0.130	0.134	0.169	0.157	0.130	0.102	0.102	0.059	0.044					
**Pop14**	0.159	0.212	0.226	0.127	0.146	0.194	0.180	0.148	0.119	0.120	0.060	0.051	0.046				
**Pop15**	0.159	0.202	0.220	0.125	0.133	0.168	0.154	0.129	0.106	0.104	0.053	0.044	0.044	0.042			
**Pop16**	0.247	0.303	0.316	0.227	0.219	0.236	0.221	0.213	0.190	0.185	0.158	0.146	0.142	0.130	0.125		
**Pop17**	0.272	0.325	0.329	0.247	0.239	0.249	0.231	0.238	0.200	0.202	0.165	0.161	0.159	0.147	0.139	0.022	
**Pop18**	0.266	0.324	0.332	0.239	0.230	0.243	0.229	0.221	0.204	0.201	0.158	0.160	0.160	0.144	0.135	0.028	0.025

### Genetic Structure and Differentiation among Populations

A highly significant (P<0.001) genetic difference was found among groups, among populations, and within populations (Table 5). The results from the AMOVA showed that 51.44% genetic variation occurred among populations (P<0.001) and the remaining 48.56% existed within populations (P<0.001). When these populations were classified into six groups based on the results of the clustering analysis, the variance among populations within the groups was 13.06%, whereas the variance among groups was 41.24%. In particular the AMOVA for the populations (Pop11, Pop12, Pop13, Pop14 and Pop15) from Group 4 according to the UPGMA tree showed that 22.0% of genetic variation occurred among populations (P<0.001) and 78.0% occurred within populations (P<0.001) (Table 4). Consistently both Nei's estimate of population substructure (*G_ST_*) and gene flow estimate (*Nm*) indicated a high level of population differentiation (*G_ST_* = 0.55, *Nm* = 0.41).

**Table 5:** AMOVA of populations and geographic groups in *E. arundinaceus* native to China.

Source of variation	d.f.	Sum of squares	Variance component	Total variance	P value
Among populations	17	3485.67	20.43	51.44%	<0.001
Within populations	146	2814.92	19.28	48.56%	<0.001
Among groups	5	2651.16	17.40	41.24%	<0.001
Among populations within groups	12	835.51	5.51	13.06%	<0.001
Within populations	146	2814.92	19.28	45.70%	<0.001

## Discussion

### Genetic Variation

In previous reports, the genetic diversity of *E. arundinaceus* was studied using individual clones, which were collected from Southeast Asia and Chinese tropical and subtropical regions. These studies showed the variation level of *E. arundinaceus* was different in different regions. The genetic diversity of *E. arundinaceus* clones in Indonesian was studied using morphological traits, demonstrating those clones had low genetic variation [14]. The result was confirmed in later experiments using other *E. arundinaceus* clones from Indonesia with rDNA, RAPD and RFLP markers [18]–[20]. Clones from India had an intermediate level of diversity [18]–[21]. The variation level of clones from the Philippines was similar to that of Indonesian clones, while the variation level of clones from Vietnam was similar to that of India clones [18]. In our study, *PPB* over 18 natural populations of *E. arundinaceus* in China was 80.0%, lower than the *PPB* value (AFLP, 99.3%) in the study of Cai et al. [18], but higher than the values (ISSR, 64.9% and RAPD, 70.1%) by Zhang et al. [16]–[17], and (AFLP, 69.2%) by Tsuruta et al. [34]. Collectively these reports revealed a high level of genetic diversity in Chinese *E. arundinaceus*.

Comparisons of the genetic variation levels of *E. arundinaceus* from the Philippines, Indonesia, India, Vietnam and China, show that *E. arundinaceus* from pacific Island countries (the Philippines and Indonesia) has lower genetic variation. In contrast, *E. arundinaceus* collections from continental countries (India, Vietnam and China) have larger genetic variation. We speculated that the low genetic variation of *E. arundinaceus* from island countries was generated by the effect of ocean isolation and relatively homogenous environments in the countries. The pacific island countries are isolated by the ocean, which may have effectively blocked or minimize gene flow from germplasm outside the islands, consequently reducing genetic diversity [35]. In the current study, the “isolation effect” was also evidenced in the genetic diversity of Chinese *E. arundinaceus* populations (Pop16, Pop17 and Pop18) from Hainan island (Hainan province) which had lower genetic variation (*PPB* = 20.56% – 25.83%, *H_E_* = 0.081 – 0.901) than the mean of all populations (*PPB* = 27.07%, *H_E_* = 0.099) from China. Possibly the germplasm on Hainan Island was isolated from receiving pollen from the germplasm on Chinese mainland by the Qiongzhou Strait. Similarly, mountains, especially the high mountains in the southwestern Chinese provinces, could form physical isolations limiting pollen facilitated gene flow among *E. arundinaceus* populations. It appears that the populations from Sichuan Basin (Pop1, Pop2 and Pop3) and those from Sichuan Daliangshan region (Pop4 and Pop5) presented a geographical differentiation separated by southern mountains of the Tibetan Plateau. Similarly, the populations from Guizhou (Pop8 and Pop9) and the populations from Guangxi (Pop11 and Pop12, except Pop10) were separated by mountains of the Yunnan-Guizhou Plateau. Those mountains might also isolate the populations in Yunnan (Pop6 and Pop7) from those in other regions. However, the populations (except Pop2 and Pop3) from these isolated regions had higher genetic variation than the mean of all populations from China, suggesting that the effect of isolation by mountains was less than from the ocean.

This is the first report characterizing genetic variation in *E. arundinaceus* through examining Chinese native populations and revealing new biological characteristics of the species. In this study, the average of within population diversity in *E. arundinaceus* (*H_E_* = 0.245) is higher than short-lived perennial (*H_E_* = 0.20), mixed-mating species (*H_E_* = 0.18) and selfers (*H_E_* = 0.12), but similar to outcrossers (*H_E_* = 0.27) reported by Nybom [36]. The results were not reported in previous reports. The *H_E_* value of *Miscanthus floridulus* (*H_E_* = 0.30) [37] was similar to the results of *E. arundinaceus* in this report, while the *H_E_* value of *Saccharum spontaneum* (*H_E_* = 0.23) [38] was lower than the value in *E. arundinaceus*. The high *He* value of *E. arundinaceus* revealed in this experiment suggests that *E. arundinaceus* be an outcrossing species.

### Genetic Structure of Populations

In this study, the Nei's estimate of *E. arundinaceus* population substructure (*G_ST_*) was 0.55, indicating more than a half of genetic variation occurred among populations. The results of *G_ST_* was similar to the results from AMOVA, which showed that 51.44% genetic variation existed among populations (P<0.001) and the remaining occurred within populations (P<0.001). Chang et al. (2012) reported genetic variation among populations was lower than that within populations in *S. spontaneum* [38]. Similar results were reported in *M. floridulus* populations [37]. Interestingly, the AMOVA of the populations from Guangxi and Guangdong (except Pop10) in this study, showed that 22.00% genetic variation occurred among populations (P<0.001) and 78.00% occurred within populations (P<0.001). As the populations are distributed in neighboring and similar environmental conditions without significant landmasses between them, gene flow among the populations may take place more frequently. Consequently, the populations do not differentiate into distinct populations. The result was more similar to the *S. spontaneum* and *M. floridulus* populations. Hamrick and Godt [39] pointed out that the genetic variation of outcrossing species occurred among populations was lower than within populations, and a similar result was found by Nybom [36]. Our study suggests that the genetic structure of *E. arundinaceus* populations is affected by the natural landforms and geographical conditions.

Gene flow (*N*m) would be able to resist the effect of genetic drift within populations and prevent the differentiation of populations as the value of *N*m >1, and when the value of *N*m <1 the genetic drift could lead to genetic differentiation among populations [40]. Outcrossing species have higher levels of gene flow [36], but the *N*m value of *E. arundinaceus* (an outcrossing species) populations in this study was only 0.41, indicating that there was a lower level of gene flow and significant genetic differentiation among the 18 populations. The natural landforms in the sampling areas of *E. arundinaceus* forming the geographic isolation and heterogeneity of the ecological environment affect gene flow, the genetic and geographical divergence among the populations [41]. Some *E. arundinaceus* populations in this study were isolated by ocean or mountain. It appears that the isolation affected not only gene flow but also the genetic diversity of *E. arundinaceus* through natural selection within local environments. In our study 18 *E. arundinaceus* populations were clustered into six groups, which belonged to different isolated regions. The Mantel tests indicated that there was no significant associated relationship between genetic distance and geographic distances between populations. The result was similar to that in *S. spontaneum* [38]. Although not statistically significant, the correlation coefficient between genetic and geographic distances may have affected the population structure, but at a magnitude less than geographic isolation.

In addition to diploids (2n = 2x = 20), most Chinese *E. arundinaceus* plants reported previously are tetraploids (2n = 4x = 40) and hexaploids (2n = 6x = 60) [15]. The altered ploidy might contribute to the genetic variation in the Chinese germplasm since gene flow between plants of altered ploidy is likely limited, consequently genetic divergence would occur. However, the geographic distribution patterns of the three ploidy forms in Chinese *E. arundinaceus* germplasm are elusive. Further investigation efforts on the association between ploidy forms and genetic variation of the native germplasm in Asian countries, especially China may shed light on the evolution and formation of genetic variability within the species.

## Supporting Information

Appendix S1 **SRAP data for 18 populations of** ***Erianthus arundinaceus*** **amplified using 20 primer pairs, coded as presence (1) and absence (0).** Note: data rows in red color were excluded in data analysis due to more than 161 of “0” and data rows in blue color were changed to monomorphic loci due to more than 161 of “1” according to Lynch and Milligan [25].
(XLS)
